# A study protocol for the cardiac effects of a single dose of either oxytocin 2.5 IU or carbetocin 100 µg after caesarean delivery: a prospective randomized controlled multi-centre trial in Norway

**DOI:** 10.12688/f1000research.73112.1

**Published:** 2021-09-27

**Authors:** Maria Bekkenes, Marte Morin Jørgensen, Anne Flem Jacobsen, Morten Wang Fagerland, Helene Rakstad-Larsen, Ole Geir Solberg, Lars Aaberge, Olav Klingenberg, Trude Steinsvik, Leiv Arne Rosseland

**Affiliations:** 1 Department of Research and Development, Division of Emergencies and Critical Care, Oslo University Hospital, Oslo, Norway; 2 Institute of Clinical Medicine, Faculty of Medicine, University of Oslo, Oslo, Norway; 3 Department of Anaesthesia, Akershus University Hospital, Lørenskog, Norway; 4 Division of Obstetrics and Gynaecology, Oslo University Hospital, Oslo, Norway; 5 Oslo Centre for Biostatistics and Epidemiology, Research Support Services, Oslo University Hospital, Oslo, Norway; 6 Department of Cardiology, Oslo University Hospital, Oslo, Norway; 7 Department of Medical Biochemistry, Oslo University Hospital, Oslo, Norway; 8 Department of Laboratory Medicine, Drammen Hospital, Vestre Viken Hospital Trust, Drammen, Norway

**Keywords:** Oxytocin, carbetocin, troponin I, anaesthesia, caesarean delivery, uterine atony

## Abstract

**Background**: Both oxytocin and carbetocin are used to prevent uterine atony and post-partum haemorrhage after caesarean delivery in many countries, including Norway. Oxytocin causes dose-dependent ST-depression, troponin release, prolongation of QT-time and arrythmia, but little is known about myocardial effects of carbetocin. We have previously demonstrated comparable vasodilatory effects of oxytocin and carbetocin and are now undertaking a Phase 4 trial to investigate whether carbetocin causes similar changes to myocardial markers compared with oxytocin.

**Methods:** Our randomized controlled trial will be conducted at three obstetrics units at Oslo University Hospital and Akershus University Hospital, Norway. Planned enrolment will be of 240 healthy, singleton pregnant women aged 18 to 50 years undergoing planned caesarean delivery. Based on pilot study data, each participant will receive a one-minute intravenous injection of either oxytocin 2.5 IU or carbetocin 100 µg during caesarean delivery. The prespecified primary outcome is the change from baseline in high-sensitive troponin I plasma concentrations at 6–10 hours after study drug administration. Secondary outcomes include uterine tone grade at 2.5 and five minutes after study drug administration, adverse events for up to 48 hours after study drug administration, estimated blood loss within eight hours of delivery, need for rescue treatment and direct/indirect costs. Enrolment and primary analysis are expected to be completed by the end of 2021.

**Discussion: **Women undergoing caesarean delivery should be assessed for cardiovascular risk particularly as women with an obstetric history of pregnancy induced hypertension, gestational diabetes mellitus, preterm birth, placental abruption, and stillbirth are at increased risk of future cardiovascular disease. Any additional ischaemic myocardial risk from uterotonic agents will need to be balanced with the benefit of reducing the risk of postpartum haemorrhage. Any potential cardiotoxicity difference between oxytocin and carbetocin will help inform treatment decisions for pregnant women.

**Registration:** Clinicaltrials.gov NCT03899961 (02/04/2019).

## Abbreviations

AE: adverse event

AHUS: Akershus University Hospital

CI: confidence interval

CTCAE: Common Terminology Criteria for Adverse Events

IU: international unit

IV: intravenous

mmHg: millimetre of mercury

OUH: Oslo University Hospital

PMI: perioperative myocardial injury

SAP: statistical analysis plan

## Introduction

Women who undergo caesarean delivery are at risk of excessive postpartum bleeding caused by uterine atony. Prophylactic administration of intravenous (IV) oxytocin to prevent uterine atony is standard first-line practice after caesarean delivery in many countries, including Norway. ^1, 2^ Nevertheless, serious cardiovascular adverse events, including ST segment depression, hypotension and tachycardia have been reported after IV oxytocin. ^2^ Women with long QT syndrome 2 have a particularly high postpartum risk of lethal arrhythmias. ^3^ Oxytocin dose reduction and/or increased infusion duration may reduce risk of some cardiac-related adverse effects and increase patient safety. ^2^

Carbetocin, a synthetic oxytocin receptor agonist with a significantly longer half-life than oxytocin, reduces postpartum blood loss by stimulating uterus contraction. ^4- 6^ Carbetocin has been in clinical use for the prevention of postpartum haemorrhage in Europe since 1999, and a room-temperature stable formulation has been available since 2015. ^7^ The efficacy is documented in several randomized controlled trials and meta-analyses. ^5, 8- 10^ Although both oxytocin and carbetocin have similar haemodynamic effects, ^11- 13^ there is insufficient evidence of their effects on the myocardium. Cardiac troponin I is a protein that is released by myocardial myocytes when starved of oxygen and can be used as an indirect measure of ischaemic heart damage. Perioperative myocardial injury can be defined as elevated or increased cardiac troponin I plasma concentration, with or without additional ischaemic signs or symptoms. ^14^ High-sensitivity detection assays can be used to monitor normal ranges of plasma troponin I concentrations. ^15^

## Aims and objectives

The aim of this Phase 4 trial is to determine any potential differences between oxytocin and carbetocin in their myocardial effects by measuring plasma troponin I using a high-sensitivity assay in healthy women with a singleton pregnancy undergoing a planned caesarean delivery. Plasma concentrations will be collected before caesarean delivery and 6–10 hours after study drug administration. Other endpoints relating to uterus tone, blood loss, blood pressure, heart rate, post-operative pain and side effects will also be assessed.

## Protocol

The study is a parallel group, randomized, patient- and investigator-blinded Phase 4 study. Enrolment of participants started in April 2019 and is anticipated to be completed by the end of 2021. The protocol has been approved by the Regional Committee for Medical Research Ethics and the Norwegian Medicines Agency, and is being conducted according to the Good Clinical Practice principles that have their origins in the Declaration of Helsinki. The trial was registered at Clinicaltrials.gov ( NCT03899961) in April 2019.

### Ethics approval and consent to participate

The project has approval from the Regional Committee for Medical and Health Research Ethics (REC **2014/1210**), The Norwegian Medicines Agency and the Institutional Data Protection officer at Oslo University Hospital. Signed informed consent form and expected cooperation of the participants for treatment and follow-up will be obtained and documented according to the International Council of Harmonisation-Good Clinical Practice (ICH GCP), and national/local regulations.

### Study setting

Women will be recruited from the general population at the three birth clinics at Oslo University Hospital (two clinics) and Akershus University Hospital in Norway.

### Eligibility criteria

All participants will have a normal singleton pregnancy at gestational age of 36 weeks or more, and will be able to read and understand Norwegian. Women with common comorbid diagnoses (diabetes, hypothyreosis, hypertension, etc) and pregnancy after *in vitro* fertilization will also be eligible for enrolment. Women will be excluded from enrolment if they have any of the following: placenta praevia or invasive placenta; pre-eclampsia; a bleeding disorder, such as von Willebrand disease type I; current treatment with low-molecular-weight heparin or other anticoagulation medication (not including aspirin); any known intolerance to either of the study drugs; prolonged QT-time or other serious cardiac disease; liver or kidney failure; epilepsy; or any medical reason why, in the opinion of the investigator, the patient should not participate.

### Recruitment

A total of 240 healthy pregnant women aged between 18 and 50 years will be included in our trial. Signed informed consent form and expected cooperation of the participants for treatment and follow-up will be obtained and documented according to the International Council of Harmonisation-Good Clinical Practice (ICH GCP), and national/local regulations. All data pertaining each enrolled participant will be entered into the electronic clinical report file (CRF; Viedoc ^®^, Uppsala, Sweden).

*Screening*

Potentially eligible participants will be screened by the principal investigator for inclusion after their last consultation before their scheduled delivery. Oral and written information about the trial will be provided to each woman at least 24 hours before delivery and written informed consent obtained before randomization. Consent, participation and redraw of consent will be documented in electronic patient record. All screened women, including those who do not give consent to participate in the study, will be registered by number. Screened women who are not enrolled due to exclusion criteria or non-fulfilment of inclusion criteria will be registered by number and the reason for not participating in the study will be recorded.

### Randomization and blinding

Participants will be randomized 1:1 to receive either carbetocin 100 μg or oxytocin 2.5 IU after caesarean delivery. The patients will be randomized according to a computer-generated list of random numbers, with block sizes unknown to the researchers as an integral part of Viedoc – the eCRF solution. Although the standard procedure according to its label is to administer 1 mL of carbetocin (100 μg/mL), ^7^ to maintain treatment masking, both study drugs will be diluted to 5 mL using normal saline by a trained member of staff otherwise uninvolved with the trial, and labelled with the trial identification and randomization number according with ICH GCP and local regulations.

### Study drug dosage and administration

A single dose of either oxytocin 2.5 IU (Syntocinon ^®^, Swedish Orphan Biovitrum, Stockholm, Sweden) or carbetocin 100 μg (Pabal ^®^, Ferring Pharmaceuticals, St-Prex, Switzerland) will be administered by the investigator (a trained anaesthetist) as a one-minute IV injection immediately after delivery of the baby’s head and shoulders. IV oxytocin 2.5 IU is standard dose used prophylactically at our institutions. Both oxytocin and carbetocin are used prophylactically after delivery to prevent uterine atony and excessive blood loss.

### Prespecified analyses

*Primary endpoint*

The primary endpoint is the difference between the oxytocin 2.5 IU and carbetocin 100 μg treatment groups in change from baseline in high-sensitive troponin I plasma concentrations 6–10 hours after study drug administration.

*Secondary outcomes*

Uterine tone will be assessed at 2.5 minutes and five minutes after study drug administration, using a numerical rating scale 0–10, where 0 = no tonus and 10 = maximum tonus, and 7 = clinically satisfactory tonus. ^13^ Blood loss will be estimated by volume during the surgical procedure as well as calculating estimated blood loss based on haematocrit percentage within 24 hours after delivery, height and weight prior to caesarean delivery. ^16^

Postoperative pain during the first 48 hours after delivery will be assessed in a subgroup of women at one centre. For these women, pain intensity (numerical rating scale 0–10) and opioid consumption (time and dose) will be recorded. In addition to the standard pain-relief treatment, these patients will have a patient-controlled intravenous morphine pump.

Direct and indirect healthcare costs will be assessed. Direct costs will include administered drugs related to prophylaxis and treatment of uterine atony, blood loss, and side effects of the therapeutic interventions. Indirect costs include the number of hours staff spend with patients in theatre and in post-anaesthesia care unit.

*Vital signs monitoring*

Vital signs and baseline blood tests (haemoglobin and sodium concentrations) of participating women will be recorded prior to administration of anaesthesia. Throughout each caesarean delivery, there will be continuous monitoring of vital signs, including echocardiogram, blood pressure and heart rate.

Routine assessments of neonatal status will be recorded (Apgar 1 and 5 minutes, umbilical vein and artery acid-base status).

### Safety assessments

The participants will be informed about the expected adverse events (AEs) prior to study enrolment and instructed to score grade of AEs at 0–2, >2–5 and >5–10 minutes after the start of study drug administration, when the majority of AEs are expected to occur. Each participant will be instructed to inform the investigator immediately if they manifest any signs or symptoms they perceive as AEs the following 48 hours (duration of the trial). Unexpected serious adverse events will be reported, also after this period, until discharge from the hospital. All AEs will be recorded according to the Common Terminology Criteria for Adverse Events version 4.0 (CTCAE).

Suspected unexpected serious adverse reactions that result in death, are immediately life-threatening, require hospitalisation, result in persistent or significant disability or incapacity, will be reported immediately to the health authorities. Other suspected serious unexpected adverse reactions will be reported in an unblinded manner to the health authorities as soon as possible but within a maximum of 15 days of first knowledge by the Sponsor (Oslo University Hospital). In order to keep the Investigator and other persons generating data to the study blinded to treatment group, the unblinding and the reporting will be performed by the clinical trial unit at Oslo University Hospital, who is also responsible for monitoring the trial and randomization.

*Expected adverse events*

Expected AEs are feeling of warmth, chest pain, shortness of breath, palpitations, flushing, headache, nasal congestion, xerostomia, and metallic taste. Both drugs in the study will lead to vasodilatation with a decrease in blood pressure and an increase in heart rate. The causal relationship of each AE to the study medication will be assessed by the investigators as either unrelated, unlikely to be related, possibly or probably related or definitely related.

### Concomitant medication

*Rescue medication*

In case of uterus atony, patients will be treated with rescue oxytocin 1 IU every 2 minutes up to maximum 5 IU. Time of rescue medication administration will be recorded. Any additional treatment required, whether medical or surgical, will be decided by the attending obstetrician and anaesthesiologist according to local guidelines.

*Anaesthesia and pain medication*

Spinal anaesthesia will be given according to study procedure (2 mL bupivacaine [5 mg/mL] + 0.4 mL fentanyl [50 μg/mL]), hypotension prophylaxis (0.5 μg/kg IV phenylephrine [0.1 mg/mL] followed by infusion rate 0.25 μg/kg/min) and IV volume (isotonic saline) 10 mL/kg starting concomitantly with spinal anaesthesia. Spinal induced hypotension (systolic arterial pressure <90 mmHg) will be treated with an extra IV bolus of phenylephrine if the heart rate is above 60 beats/min or with IV ephedrine 5–10 mg if 60 beats/min or below. Analgesic medication will be administered as required by the participants and will include oral paracetamol 1 g and oral ibuprofen 400–600 mg four times per day. Patients may be given one IV bolus ketorolac trometamol 30 mg and IV bolus or oral oxycodon administered according to local guidelines and as required by the participants.

All medication interventions, including drug dose and time of administration will be recorded.

### Criteria for patient discontinuation

Patients may be discontinued from study treatment and assessments at any time. Specific reasons for discontinuing a patient for this study are as follows: (1) Voluntary discontinuation by the patient who is at any time free to discontinue her participation in the study, without prejudice to further treatment; (2) Severe non-compliance to protocol as judged by the principal investigator; (3) Incorrect enrolment i.e., the patient does not meet the required inclusion/exclusion criteria for the study. Patients who withdraw or are withdrawn from the study, will stop further treatment. Reasons for discontinuation will be documented.

### Laboratory tests

Blood samples from participating women will be collected at baseline (prior to caesarean delivery) and between six and 10 hours after study drug administration. Haemoglobin and sodium concentrations will be assessed using the point-of-care blood gas analyser(s) (ABL 800 Analyzer ^®^, Radiometer, Copenhagen, Denmark). Blood plasma samples, stored in a certified research biobank freezer (–70 °C), will be analysed centrally for troponin I concentration as one batch at the Vestre Viken Trust, Drammen Hospital, Norway, once the last participant has completed the study. Plasma Troponin I concentrations for each participant will be measured using high-sensitive detection with a chemiluminiscent microparticle immunoassay (Alinity i ^®^, Abbott, Illinois, U.S.A.). Normal values of troponin I in a female population (age 18 to 50 years) should be <15 ng/L, with the cut-off corresponding to the 99 percentile of a healthy reference population. ^17^

### Data collection and monitoring

Every investigator responsible for entering data into the eCRF will be provided with a unique identity and password, thereby creating an electronic signature of the investigator attesting the accuracy of the data on each eCRF. If any assessments are omitted, the reason for such omissions will be noted on the eCRFs. Corrections, with the reason for the corrections if applicable, will be dated and signed by the investigator in the eCRF. Electronic files, including the data from CRF entered into an electronic database software will be stored at the research server at Oslo University Hospital. This data storage is administered by the Data Inspectorate’s local representative. The patient identification and the code list will be kept by the principal investigator in a locked office, ensuring confidentiality. Investigators involved with data analysis will have access to the study data base. Patient files will be kept for the maximum period of time permitted by each hospital.

The Clinical Study Monitor, independent from the investigators, will visit each investigator regularly to will check and collect completed CRFs, discuss the progress of the study and monitor drug usage according to ICH GCP guidelines. The monitoring will also include source data verification (data audit). A data monitoring committee will not be used as the data collection is expected to be straightforward. Authorized representatives of a regulatory authority and Ethics Committee may visit the centre to perform inspections, including source data verification.

### Power and sample size considerations

The sample size required for our current study was calculated using data from a pilot study of 40 patients, in which the largest difference in plasma troponin I concentration was found at 10 hours, with a mean ± standard deviation change from baseline of 0.41 ± 0.79 ng/L in the carbetocin group versus 1.78 ± 4.48 in the oxytocin group. The sample size calculation was thus based on 80% power to detect a between-group difference in change from baseline to 10 hours of 1.37, to be analysed using a two-sample T-test with adjustments for unequal variances. With a significance level of 5%, our study will need to include 178 patients (89 in each treatment group) in this confirmatory trial. To adjust for loss of information from missing values and patient drop outs, 240 women will be enrolled. The drop-out rate after enrolment is expected to be low as the duration of the study is short.

### Analysis plan

The primary endpoint and all other continuous endpoints that include baseline and ≥1 follow-up measurement(s) will be analysed with linear regression models, with the follow-up measurement defined as the dependent variable and treatment group and baseline measurement defined as independent variables. Based on the fitted models, we will estimate treatment group differences in changes from baseline with 95% confidence intervals (CIs), together with a p-value for the null hypothesis of no treatment group difference. We expect at least some degree of skewness in the primary and some of the secondary endpoints, and maybe in the residuals from the linear regression models. The amount of skewness will be assessed with histograms and descriptive statistics, such as mean, median, variance, and the skewness index. In cases where the distribution of the residuals deviates markedly from the normal distribution, or when the endpoints themselves are too skewed to use means as measures of central tendency, we will use median regression models instead of linear regression models, thus analysing between-group differences in median changes from baseline instead of mean changes from baseline. Standard errors and CIs in the median regression models will be obtained via bootstrapping with 100 replications.

Continuous endpoints measured at a single time point will be analysed with two-sample T tests and 95% CIs for the difference between means based on the t distribution, with adjustment for unequal variances. Median regression will be used for variables with highly skewed distributions.

Binary outcomes will be analysed with Fisher mid-P tests and Newcombe hybrid score confidence intervals for the difference between probabilities. ^18^ Ordered categorical outcomes will be analysed with score tests for effect in a proportional odds model (the Wilcoxon-Mann-Whitney test). ^18^

All analyses will be performed on the intention-to-treat population. AEs and vital signs will be presented with descriptive statistics. In addition, the primary endpoint will also be analysed using the per protocol population. Further details of the statistical methods, including definitions of the intention-to-treat and per protocol populations, how to handle missing data, and sensitivity and exploratory analyses are available in the Statistical Analysis Plan (SAP) available on the trial registration page.

### Data dissemination plan

The results of the study will be published in an open-access peer-reviewed journal and deposited at the trial registration page. Requests for data sharing/case pooling may be directed to the project Principal Investigator: Professor Rosseland on email: lrossela@ous-hf.no.

### Study status

The trial is actively recruiting at all sites.

## Discussion

Both oxytocin and carbetocin are used routinely after caesarean delivery to prevent uterine atony and excessive blood loss. ^5, 8^ We know that oxytocin causes dose-dependent ST-depression, troponin release, prolongation of QT-time and arrythmia, but little is known about myocardial effects of carbetocin. ^19^ Our ongoing study has been designed to assess high-sensitivity troponin I plasma concentrations after the prophylactic administration of either oxytocin or carbetocin to determine whether there are any differences in their myocardial effects. The protocol of this parallel, randomized, blinded phase 4 study has been approved by the Regional Committee for Medical Research Ethics and the Norwegian Medicines Agency, and follows the principles for Good Clinical Trial practice. In addition, the biobank has been approved by the Regional Committee for Medical Research Ethics for storage of blood plasma from the participating women. All enrolled women will have the same techniques performed for their caesarean delivery, comprising a Pfannenstiel skin incision. Once the baby and placenta have been delivered, exteriorization of the uterus will be performed according to local guidelines. This will limit confounding variables of different surgical techniques interference with assessment of uterine tone. The primary endpoint of change in troponin I (high-sensitive detection method) will be analysed after the final participant has completed the trial as one batch, therefore the results will not influence any participant’s treatment, including rescue treatment with oxytocin, or follow-up.

Although the use of oxytocin has reduced the risk of postpartum haemorrhage, its haemodynamic effects may cause myocardial ischaemia. ^20^ Carbetocin, a synthetic derivate of oxytocin but with a longer half-life (median terminal elimination half-life is 33 minutes after intravenous administration and 55 minutes after intramuscular administration ^7^) shows similar haemodynamic effects to oxytocin. ^11- 13^ Like oxytocin, carbetocin binds to the oxytocin receptor therefore affects the same tissues. ^7^ Oxytocin has been shown to increase cardiac output by decreasing vascular tone in small and peripheral arteries, resulting in a lower blood pressure and a compensatory increase in heart rate, implying increased myocardial oxygen demand. ^21^ Carbetocin also increases cardiac output. ^13^ Minor differences have been detected in the recovery times for heart rate and blood pressure changes, with heart rate elevations lasting slightly longer with carbetocin, possibly related to the longer half-life of carbetocin compared with oxytocin. ^11, 13^

Perioperative myocardial injury (PMI) is an important but often undetected complication on noncardiac surgery as it rarely is accompanied by typical symptoms of myocardial ischaemia, such as chest pain, or dyspnea. ^14^ Although PMI is common after noncardiac surgery (approximately 16% of patients with a median age of 74 years), ^14^ little is known about the risk of PMI in women undergoing caesarean delivery. One small study of 26 women showed troponin I plasma concentrations suggestive of myocardial ischaemia 12-hours post caesarean delivery. ^22^ All 26 women had received postpartum intravenous oxytocin (10 IU, over 30 seconds), ^22^ which is a much higher dose than our current study, in which women will be randomized to receive a one-minute intravenous infusion of either oxytocin 2.5 IU or carbetocin 100 μg. Nevertheless, the clinical relevance is that PMI appears to increase post-operative mortality risk. In a prospective cohort study of nearly 22,000 people undergoing in-patient noncardiac surgery, a peak postoperative high sensitivity troponin T increase of at least 5 ng/L during the first three days after surgery was significantly associated with 30-day mortality even if there were no ischaemic signs or symptoms (adjusted hazard ratio, 4.69; 95% CI, 3.52–6.25). ^23^ A smaller study of 2018 patients undergoing nearly 3000 surgical procedures also showed that a preoperative to postoperative increase of ≥14 ng/L of high-sensitivity troponin T (detected in 285 patients) was associated with increased mortality at 30-days (9.8% of patients with PMI versus 1.6% without PMI) and at one-year (22.5% of patients with PMI versus 9.3% without PMI). ^14^ Our study will assess high-sensitivity troponin I levels as we expect very small levels of troponin release in response to either oxytocin or carbetocin. Currently, the Abbott troponin I immunoassay has a lower limit of detection of 0.1 ng/L whereas the Roche troponin T immunoassay has a higher lower limit of detection of 5.0 ng/L. ^24^ Our baseline assessments will help provide a reference range of troponin I for pregnant women at term. All troponin I measurements below 0.1 ng/L will be designated as 0 ng/L.

### Strengths and limitations

The study design is robust, the sample size based on *a priori* power calculations with parameters estimated from recent, relevant pilot data, and the trial will be conducted in a representative sample of pregnant women at three Norwegian birth clinics. Our study is enrolling women from general obstetric practice, including those with comorbidities such as gestational diabetes and hypertension. There is no upper limit for body mass index for participating women, and with an upper age limit of 50 years, nearly all women with singleton pregnancies at the three clinics will be eligible for inclusion. Women aged between 18 and 50 years make up approximately 25% of the Norwegian population. ^25^ Although this is not an international study, many other countries in Europe have similar obstetric practices.

In 2019, a core outcomes set was published for prevention of post-partum haemorrhage to aid better comparison of clinical trials ensuring a minimum of outcomes to be reported. ^26^ Our protocol was approved prior to its publication. Nevertheless, our study will assess seven out of nine of the core outcomes (blood loss, shock, transfer to higher level of care, use of additional haemostatic interventions and adverse events). We will not assess breastfeeding or patient satisfaction with treatment, but will report pain and pain relief medication.

All caesarean deliveries will be performed with standardized anaesthesia and surgery, including the meticulous blood pressure control to reduce cardiac troponin I due to spinal anaesthesia–induced hypotension and/or tachycardia. Both intraoperative tachycardia and hypotension are associated with myocardial injury after noncardiac surgery, ^27^ thereby, we are limiting an important confounding factor that may complicate the interpretation of our results.

As all participating women have to be able to read and understand Norwegian in order to provide informed consent, this may limit generalizability to diverse ethnic background to some extent. We will provide detailed demographic and baseline characteristics of all participating women.

In the analyses of direct and indirect costs, any potential drug specific long-term cardiac adverse events rate will not be included. We expect differences in health care costs between oxytocin and carbetocin cost to be minor.

### Interpretation

Women with an obstetric history of pregnancy induced hypertension, gestational diabetes mellitus, preterm birth, placental abruption, and stillbirth are at increased risk of future cardiovascular disease. ^28, 29^ Women undergoing caesarean delivery should be assessed for cardiovascular risk. Any additional ischaemic myocardial risk from uterotonic agents administered postpartum need to be balanced with the benefit of reducing the risk of postpartum haemorrhage. We anticipate that data from our study will help future clinical management decisions around planning delivery in pregnant women, including those with heart disease. As far as we can tell, no other study has compared the myocardial effects of oxytocin and carbetocin when used as postpartum uterotonic agents after caesarean delivery. We currently know little about troponin release after vaginal delivery and the role of uterontonics on troponin release in this population, which would require further investigation.

## Conclusion

The results of our trial will help inform treatment decisions around preventing uterine atony in women undergoing caesarean delivery.

## Data availability

### Extended data

Zenodo: Supporting material for article ‘A study protocol for the cardiac effects of a single dose of either oxytocin 2.5 IU or carbetocin 100 μg after caesarean delivery: a prospective randomized controlled multi-centre trial in Norway’, https://doi.org/10.5281/zenodo.5217789. ^30^

This project contains the following extended data:

- Ethics approval CMT2014 REC English translation.pdf
- Norwegian Medicines Agency approval SLV-godkjenning CMT-studien English included.pdf
- Pasientinformasjon - v3.pdf
- Protocol v8 signed.pdf

### Reporting guidelines

Zenodo: SPIRIT and TIDieR checklists for ‘A study protocol for the cardiac effects of a single dose of either oxytocin 2.5 IU or carbetocin 100 μg after caesarean delivery: a prospective randomized controlled multi-centre trial in Norway’, https://doi.org/10.5281/zenodo.5519494. ^30^

Data are available under the terms of the Creative Commons Attribution 4.0 International license (CC-BY 4.0).
