# Supplementary material for: A study protocol for the cardiac effects of a single dose of either oxytocin 2.5 IU or carbetocin 100 µg after caesarean delivery: a prospective randomized controlled multi-centre trial in Norway
Source: F1000Res. 2022 Apr 14;10:973. Originally published 2021 Sep 27. [Version 2] doi: 10.12688/f1000research.73112.2 (PMC8561611; doi:10.12688/f1000research.73112.2)
Supplement: Peer review history

# Peer review: Reviewer response for version 1

**DOI:** 10.5256/f1000research.76738.r96674

**Published:** 2021-11-01

**Authors:** Kim Ekelund, Kim Lindelof

**Affiliations:** 1 Department of Obstetrics, Juliane Marie Centre, Rigshospitalet, Copenhagen, Denmark; 2 Department of Anesthesiology, Juliane Marie Centre, Rigshospitalet, Copenhagen, Denmark

**Review #2**

 Thank you for the opportunity to review this manuscript.

 The main objective of this study was to compare the cardiac effect, measured as the increased level of Troponin I, after intravenous administration of Oxytocin 2.5IU or Carbetocin 100µg during cesarean section (CS) in healthy, singleton pregnant women aged 18 to 50 years undergoing planned caesarean. The prespecified primary outcome was change from baseline in high-sensitive troponin I plasma concentrations at 6–10 hours after study drug administration. Secondary outcomes include uterine tone grade at 2.5 and five minutes after study drug administration, adverse events for up to 48 hours after study drug administration, estimated blood loss within eight hours of delivery, need for rescue treatment, and direct/indirect costs.

**Overall**, the manuscript is well written, easy to read, and thoroughly describes a protocol for a Norwegian potentially clinically relevant RCT.

 I have a few general comments. The protocol is registered on Clinicaltrials.gov as NCT03899961 and is referred to as Carbetocin Myocardium Trial 2014 Part 2 (CMT2014/2). The authors have also registered The Clinical Carbetocin Myocardium Trial (CMT) as NCT02528136.

 This study is in the manuscript described as a pilot study including 40 patients. Moreover, the is a study funded by the manufacturer of Carbetocin: Ferring Pharmaceuticals, including funding of medical writing support for this manuscript. Although it is stated “The funders had no role in study design, data collection, and analysis, decision to publish, or preparation of the manuscript”, I will as a reader be more skeptical compared to a non-funded protocol. However, the protocol describes this openly and no conflicts of interest can be identified.

 Finally, I understand the study already are up and running. (“Study status: The trial is actively recruiting at all sites.”, and on Clinicaltrials.gov: September 30, 2021, is registered as Final data collection date for primary outcome measure). This makes me wonder, why I am asked to review a protocol now, and not in 2019 before recruitment began?

 Just more wondering: Has the cardiac effect of Oxytocin / Carbetocin been studied in an animal model? Has it been studied in non-pregnant adults? Can Troponin I be measured after physical activity (walking staircases, jogging, or similar?

 I have more **additional specific comments and questions:**

**“Protocol. **The study is a parallel-group, randomized, patient- and investigator-blinded Phase 4 study. Enrolment of participants started in April 2019 and is anticipated to be completed by the end of 2021…”

- Does this mean, that we are reviewing a protocol already in action?

**“Eligibility criteria. **All participants… (but not) prolonged QT-time or other serious cardiac diseases.

- Do you do a pre-CS echocardiography before inclusion? Or just during CS?

“ **Randomization and blinding. **Participants will be randomized 1:1… to a computer-generated list of random numbers, with block sizes unknown”…

- Can you provide us with a more detailed description of the randomization? It sounds like a Viedoc@ is a black box? No description can be found regarding the “integral part of Viedoc – the eCRF solution”.

**“Study drug dosage and administration **A single dose of either Oxytocin 2.5 IU (Syntocinon®, Swedish Orphan Biovitrum, Stockholm, Sweden) or Carbetocin 100 μg … will be administered by the investigator (a trained anesthetist) as a one-minute IV injection.

- Administering 2.5 IU of Oxytocin as a one-minute injection seems to be fast, and faster than the recommendations. We recommend an injection of 1IE/minute, or slower.
- Administering 100mikrog carbetocin diluted to 5ml as a one-minute iv injection also seems too fast. Should be administered slower than 1 minute.
- If the approximate ED95 for Oxytocin for elective CS is 0.35 IU and 15 µg for Carbetocin, shouldn't we test these dosages?
- In Ref 4 (Widmer *et al.*) ^1^ Oxytocin 10IU with Carbetocin 100µg is compared, They find 0.04 vs 0.09% that complains of chest pain, respectively, i.e., after a smaller dose of Carbetocin compared to what is used in this study. Have the authors considered reducing the Carbetocin dose?

**“Prespecified analyses**. *Secondary outcomes *Uterine tone will be assessed at 2.5 minutes and five minutes after study drug administration, using a numerical rating scale 0–10, where 0 = no tonus and 10 = maximum tonus, and 7 = clinically satisfactory tonus.”

- Do the obstetricians get any training in using this scale? Do they have a Gold Standard to compare with?

“Postoperative pain during the first 48 hours after delivery will be assessed in a subgroup of women at one centre.”

- Why is this relevant? And if relevant, why is this only done in one centre?

“Direct and indirect healthcare costs will be assessed”.

- Will the costs of 10 IU Oxytocin (approx. 2 €) and 100 µg Carbetocin (approx. 40€) be included in the assessment?

**“Concomitant medication** *Rescue medication *In case of uterus atony, patients will be treated with rescue oxytocin 1 IU every 2 minutes up to maximum 5 IU.”

- Is it max 5 IU in total (ie 2.5 + 1 + 1 + 0.5 ??) or is it in addition to the 2.5?
- What happens after the maximum 5 IU is administered and there still is some atony?
- Will the additional Oxytocin not disturb the picture when comparing to Carbetocin – are these women excluded from getting Troponin I measured after 10 hours?

**“Laboratory tests.**…Normal values of troponin I in a female population (age 18 to 50 years) should be <15 ng/L, with the cut-off corresponding to the 99 percentile of a healthy reference population. 17”

- In ref. 17 (Bahadur *et al*) ^2^, the Pakistan population had a median age of the included female on 56 years old with the oldest being 86 and youngest being 21 years old. Most of the people 246 (82.3%) were above 40 years old. This means that they were older than the Norwegian CS population, and Troponin I increase with age, according to ref. 15, Venge *et al*. ^3^ Do the authors have any comments on this?

- Do we know if the Pakistan women have a similar Troponin level as Norwegian?
- What was the baseline level in the pilot study?
- Is Troponin I the best measurement for myocardial injury?
- What about proBNP?
- If all the levels are < the 99 ^th^ percentile i.e. normal, can we then state that Carbetocin and Oxytocin are safe (regarding cardiac side-effects)?
- If there are minor differences but still within normal ranges, then this might not be clinically relevant? Like studies showing significant difference in blood loss on i.e., 178mL between treatment A and B... Significant - yes. Relevant - no!  Any comments?

**“Power and sample size considerations. **The sample size required for our current study was calculated using data from a pilot study of 40 patients, in which the largest difference in plasma troponin I concentration was found at 10 hours, with a mean +/- standard deviation change from baseline of 0.41+/-0.79 ng/L in the Carbetocin group versus 1.78+/-4.48 in the Oxytocin group”.

- I admit I am not used to doing statistical analysis, but does this mean that the change from the baseline can be 0.4ng/L – 0.8ng/L = -0.4ng/L? Because then Carbetocin improves the cardiac effect, reducing the Troponin I level? And similar for Oxytocin?
- Or does this mean that the data is skewed? And is “Median” not preferred instead of “Mean”?
- What is the baseline for Oxytocin and Carbetocin, respectively?
- Is this because the authors suspect the results to be clinically not relevant, although detecting significant differences?
- And is this because Ferring is funding the project? (As I mentioned in the beginning, the reader should be more suspicious).

**Analysis plan**

- I believe I do not have enough knowledge to comment relevantly on the analysis plan.  

**Strength and Limitations. **In 2019, a core outcome set was published for the prevention of postpartum hemorrhage to aid better comparison of clinical trials ensuring a minimum of outcomes to be reported…We will not assess breastfeeding or patient satisfaction with treatment, but will report pain and pain relief medication.”

- Why is this relevant to the study?
- Is the dose of Oxytocin 2.5IU used elsewhere?
- The cardiovascular events that are described after oxytocin iv dose (ST-changes, hypotension, tachycardia), have they been found after 2.5 IU?
- Will data be released at the person level (for i.e. reviewer) in case of potential cardiac damage, and high Troponin I level during the CS?

Is the study design appropriate for the research question?

Yes

Is the rationale for, and objectives of, the study clearly described?

Partly

Are sufficient details of the methods provided to allow replication by others?

Partly

Are the datasets clearly presented in a useable and accessible format?

Not applicable

Reviewer Expertise:

Obstetric anesthesiology

We confirm that we have read this submission and believe that we have an appropriate level of expertise to confirm that it is of an acceptable scientific standard.

## References

[1] : Heat-Stable Carbetocin versus Oxytocin to Prevent Hemorrhage after Vaginal Birth. *N Engl J Med* .2018;379(8) : 10.1056/NEJMoa1805489 743-752 10.1056/NEJMoa1805489 29949473

[2] : Determination of high sensitive cardiac troponin I 99th percentile upper reference limits in a healthy Pakistani population. *Pak J Med Sci* .36(6) : 10.12669/pjms.36.6.2328 1303-1307 10.12669/pjms.36.6.2328 32968398PMC7501037

[3] : Normal plasma levels of cardiac troponin I measured by the high-sensitivity cardiac troponin I access prototype assay and the impact on the diagnosis of myocardial ischemia. *J Am Coll Cardiol* .2009;54(13) : 10.1016/j.jacc.2009.05.051 1165-72 10.1016/j.jacc.2009.05.051 19761938

# Response

**Published:** 2022-03-31

**Authors:** Leiv Arne Rosseland

**Affiliations:** Oslo University Hospital, Norway

*01 Nov 2021 | for Version 1*

**Kim Ekelund**, Department of Obstetrics, Juliane Marie Centre, Rigshospitalet, Copenhagen, Denmark  

**Kim Lindelof**, Department of Anesthesiology, Juliane Marie Centre, Rigshospitalet, Copenhagen, Denmark 

 Reviewer comments are in *italicized *text. Author responses are in **bold** text.

***Comment***

*Thank you for the opportunity to review this manuscript.*

*The main objective of this study was to compare the cardiac effect, measured as the increased level of Troponin I, after intravenous administration of Oxytocin 2.5IU or Carbetocin 100µg during cesarean section (CS) in healthy, singleton pregnant women aged 18 to 50 years undergoing planned caesarean. The prespecified primary outcome was change from baseline in high-sensitive troponin I plasma concentrations at 6–10 hours after study drug administration. Secondary outcomes include uterine tone grade at 2.5 and five minutes after study drug administration, adverse events for up to 48 hours after study drug administration, estimated blood loss within eight hours of delivery, need for rescue treatment, and direct/indirect costs.*

*Overall, the manuscript is well written, easy to read, and thoroughly describes a protocol for a Norwegian potentially clinically relevant RCT.*

*I have a few general comments. The protocol is registered on Clinicaltrials.gov as NCT03899961 and is referred to as Carbetocin Myocardium Trial 2014 Part 2 (CMT2014/2). The authors have also registered The Clinical Carbetocin Myocardium Trial (CMT) as NCT02528136.*

*This study is in the manuscript described as a pilot study including 40 patients. Moreover, the is a study funded by the manufacturer of Carbetocin: Ferring Pharmaceuticals, including funding of medical writing support for this manuscript. Although it is stated “The funders had no role in study design, data collection, and analysis, decision to publish, or preparation of the manuscript”, I will as a reader be more skeptical compared to a non-funded protocol. However, the protocol describes this openly and no conflicts of interest can be identified.*

**Author response**

**Studies require funding and we have been transparent about where our funding has come from. We are ensuring that the safety profiles in relation to the release of cardiac markers of two commonly used drugs are characterized fully so that clinicians will be able to make informed treatment decisions. We will report the results of this trial so that clinicians will be able to use our findings in order to improve standard of care for all women requiring prophylaxis treatment to prevent post-partum haemorrhage.**

**Our pilot study manuscript is currently undergoing peer-review at another journal.**

***Comment***

*Finally, I understand the study already are up and running. (“Study status: The trial is actively recruiting at all sites.”, and on Clinicaltrials.gov: September 30, 2021, is registered as Final data collection date for primary outcome measure). This makes me wonder, why I am asked to review a protocol now, and not in 2019 before recruitment began?*

**Author response**

**We decided to write a protocol manuscript describing the Carbetocin Myocardium Trial 2014 part 2 (CMT 2) in January 2021 when we started writing our manuscript for the Pilot study. Our rationale for publishing the protocol is to increase awareness that oxytocin and carbetocin may have slightly different effects on the myocardium. At the point we decided to publish the protocol in more detail we had already started enrollment. The enrollment progress has been delayed due to the COVID-19 pandemic. Ideally this protocol article would be published prior to recruiting patient. We hope that readers will find the protocol and intention of our clinical trial valuable when making assessments of which uterotonic agent to use.**

***Comment***

*Just more wondering: Has the cardiac effect of Oxytocin / Carbetocin been studied in an animal model? Has it been studied in non-pregnant adults? Can Troponin I be measured after physical activity (walking staircases, jogging, or similar?*

**Author response**

**The potentially harmful effects of oxytocin on heart tissue and circulation have been known for decades. The risk of such effect can to some extent be reduced by lowering the dose and increasing duration of IV injection. Notwithstanding, oxytocin receptor agonists are the recommended drug of choice for prevention and treatment of PPH. Indeed, PPH remains the leading cause of maternal death. Carbetocin has gained popularity during the recent years due to the prolonged duration of action. To our knowledge, little data exist on the effect of carbetocin on the myocardium in animal models. As both oxytocin and carbetocin are used daily at the described doses in obstetric units all over the world, we find it compelling to investigate whether the myocardial effects of oxytocin and carbetocin are comparable in healthy women undergoing ceasarean delivery of their baby, even if the myocardial effects of carbetocin are not well described in animal models. We have amended the last paragraph of the Introduction to state more clearly that although there are no animal models, the myocardial effects of oxytocin and carbetocin warrant further investigation.**

**Although animal models are lacking, there are studies that assessed the effects of oxytocin on heart tissue in pregnant women. The effect of oxytocin was studied in a clinical trial conducted by Svanström et al., including women undergoing cesarean delivery, investigating differences in the effect of oxytocin 10 IU and methylergometrine 0.2 mg on ECG changes related to myocardial ischemia. Clinically relevant occurrence of ECG changes were found in the oxytocin group, but not in the methylergometrine group. A control group of 10 non-pregnant patients receiving 10U of oxytocin also developed similar ECG changes. The authors conclude that the effects are related to oxytocin administration and not to pregnancy, surgical procedure, delivery, or sympathetic block from spinal anaesthesia** **(Svanström et al. 2008)** **.**

**The clinical relevance of transient troponin release is still unknown. High-sensitive troponins, both troponin I and troponin T, can be measured after physical activity like  prolonged endurance activities as marathon but also after short-term and intermittent exercise like 30min of running and basketball** **(Gresslien and Agewall 2016)** **. There is some uncertainty on the clinical implication of these transient troponin release. However, there is increasing concern that exercise-induced increases in troponin may not be a benign physiological response to exercise, but an early marker of future mortality and cardiovascular events** **(Aengevaeren et al. 2019)** **.**

**We have amended the manuscript (second paragraph of the ‘Discussion’) to include the above information.**

**Comment**

*I have more additional specific comments and questions:*

*“Protocol. The study is a parallel-group, randomized, patient- and investigator-blinded Phase 4 study. Enrolment of participants started in April 2019 and is anticipated to be completed by the end of 2021…” *

- *Does this mean, that we are reviewing a protocol already in action?*

**Author response**

**Yes, we have started enrollment of the study. Please see the response to a similar comment above.**

***Comment***

*“Eligibility criteria. All participants… (but not) prolonged QT-time or other serious cardiac diseases. *

- *Do you do a pre-CS echocardiography before inclusion?* *Or just during CS?*

**Author response**

**In our clinic screening the patients with electrocardiogram is not a routine part of the preparation of patients for caesarean delivery. Patients with known cardiac disease or prolonged QT-time will be considered non-eligible for study participation. Thus, the participants are not screened with a pre-CS echocardiography to be included in the study** *.* **We have added that ECG was not part of the screening process to the section ‘Eligibility criteria’.**

***Comment***

*“Randomization and blinding. Participants will be randomized 1:1… to a computer-generated list of random numbers, with block sizes unknown”… *

- *Can you provide us with a more detailed description of the randomization? It sounds like a Viedoc@ is a black box? No description can be found regarding the “integral part of Viedoc – the eCRF solution”.*

**Author response**

**We have amended the article to include a better description of the randomization process.**

***Comment***

*“Study drug dosage and administration A single dose of either Oxytocin 2.5 IU (Syntocinon®, Swedish Orphan Biovitrum, Stockholm, Sweden) or Carbetocin 100* *μ* *g … will be administered by the investigator (a trained anesthetist) as a one-minute IV injection. *

- *Administering 2.5 IU of Oxytocin as a one-minute injection seems to be fast, and faster than the recommendations.* *We recommend an injection of 1IE/minute, or slower.*
- *Administering 100mikrog carbetocin diluted to 5ml as a one-minute iv injection also seems too fast.* *Should be administered slower than 1 minute.*

**Author response**

**Administration of oxytocin analogues is performed with great variety both nationally and internationally. There has been a shift from bolus dosing toward short infusion in case of IV administration. Thomas et al. showed that 5U oxytocin as a rapid bolus (approximately 1 second) induce more powerful hemodynamic effects compared to 5U administered as a 5-minute infusion** **(Thomas et al. 2007)** **. However, little data exist comparing a 1-minute injection with a 5-minute infusion.**

**In our clinic the current practice is to administer either oxytocin 2.5 IU or carbetocin 100 µg as a 1-minute IV infusion. This is well within the recommended dosage regimen suggested in the guidelines published by Heesen et al in Anaesthesia in 2019, recommending that doses of oxytocin up to 3 IU or carbetocin up to 100** **m** **g should be administered over a time period of more than 30 seconds** **(Heesen et al. 2019)** **. Moreover, Dell-Kuster et al proved no advantage on haemodynamic stablity of administering 100** **m** **g Carbetocin IV as a 5-minute infusion compared to a 1-minute injection** **(Dell-Kuster et al. 2017)** **.**

**We have amended the article to include a statement that the duration of the injection of the study drugs was in line with current guidelines.**

***Comment***

- *If the approximate ED95 for Oxytocin for elective CS is 0.35 IU and 15 µg for Carbetocin, shouldn't we test these dosages?*

**Author response**

**It is well known that ED95 (the dose required to achieve the desired effect in 95% of the population) for oxytocin analogues are well below doses used in clinical practice for prevention of PPH in elective caesarean delivery. We still think it is important to investigate the difference in haemodynamic and myocardial effects of oxytocin and carbetocin at doses used in current clinical practice. Comparing myocardial effects of 0.35 IU of oxytocin and 15** **m** **g of carbetocin will be an interesting topic for further studies. We have not made any amendments with regard to this comment.**

***Comment***

- *In Ref 4 (Widmer et al.) Oxytocin 10 IU with Carbetocin 100 µg is compared. They find 0.04 vs 0.09% that complains of chest pain, respectively, i.e., after a smaller dose of Carbetocin compared to what is used in this study.* *Have the authors considered reducing the Carbetocin dose?*

**Author response**

**To our knowledge, Widimer et al. are comparing the effect of oxytocin 10 IU administered intramuscularly (IM) with carbetocin 100** **m** **g IM for the prevention of PPH after vaginal delivery. The dose of 100** **m** **g of carbetocin is identical to the dose used in our study, the only difference is the route of administration, IV instead of IM. Widimer et al. found that 0.04% of patients in the oxytocin group complained of chest pain compared to 0.09% in the carbetocin group, however the group differences were not significant. We have not made any amendments with regard to this comment.**

***Comment***

***“Prespecified analyses*** *. Secondary outcomes Uterine tone will be assessed at 2.5 minutes and five minutes after study drug administration, using a numerical rating scale 0–10, where 0 = no tonus and 10 = maximum tonus, and 7 = clinically satisfactory tonus.” *

- *Do the obstetricians get any training in using this scale? Do they have a Gold Standard to compare with?*

**Author response**

**The obstetricians are presented with the 11-point numerical rating scale of uterine tone prior to the operation. A similar scale has been used in several clinical studies to determine level of uterine tone numerically** **(Sarna et al. 1997, Rosseland et al. 2013, Tabl et al. 2019)** **. In our experience a uterine tone of 6 or less correlate well with the need for rescue uterotonics. Recently Cole et al. published an article concluding that the 0 to 10 numerical rating scale for uterine tone may be a reliable, standardized tool for research in reporting degree of uterotonic contraction during cesarean delivery** **(Cole et al. 2021)** **. We have added a statement to the article to include the Cole et al 2021 citation in the section ‘Secondary outcomes’**

***Comment***

*“Postoperative pain during the first 48 hours after delivery will be assessed in a subgroup of women at one centre.” *

- *Why is this relevant? And if relevant, why is this only done in one centre?*

**Author response**

**Group differences in reported pain and opioid consumption will be further investigated in a sub-study including 80 patients. We have amended the manuscript to make it clearer that there is a pain sub-study. 80 patients were deemed sufficient to see if there is a difference in post-operative pain experienced by participating women. Pain experienced by people is very subjective, and many factors affect the levels of pain a person may experience. We decided to conduct the pain sub-study just in one centre to remove as many confounding variables as possible. If the 80 women in the sub-study were distributed across the three centres, we would not be sure whether there were other external factors at play, not just the study drugs.**

****

***Comment***

*“Direct and indirect healthcare costs will be assessed”. *

- *Will the costs of 10 IU Oxytocin (approx. 2 €) and 100 µg Carbetocin (approx. 40€) be included in the assessment?*

**Author response**

**For estimation of direct and indirect healthcare costs, the costs of oxytocin and carbetocin will be included in the assessment along with the costs of rescue uterotonics, need for transfusions and duration of patient occupation of the operating room and the post-anaesthesia care unit after administration of study drug. We have made some minor edits to the article to clarify the direct and indirect healthcare costs.**

*Comment*

***“Concomitant medication **Rescue medication In case of uterus atony, patients will be treated with rescue oxytocin 1 IU every 2 minutes up to maximum 5 IU.” *

- *Is it max 5 IU in total (ie 2.5 + 1 + 1 + 0.5 ??) or is it in addition to the 2.5?*
- *What happens after the maximum 5 IU is administered and there still is some atony?*
*  *
- *Will the additional Oxytocin not disturb the picture when comparing to Carbetocin – are these women excluded from getting Troponin I measured after 10 hours?*

**Author response**

**Rescue medication with 1 IU of oxytocin for treating uterine atony will be administered every 2 minutes up to a maximum of 5 IU, in addition to either study drug (2.5 IU oxytocin or 100** **m** **g carbetocin). In case there is still a need for additional uterotonic treatment, this will be provided according to departmental procedures, including administration of prolonged oxytocin infusion, methylergometrine, prostinphenem, misoprostol or surgical or mechanical intervention as required. We have amended the article under the section ‘Rescue medication’ to clarify this point.**

**The analysis of group differences in troponin I release will be performed in the modified intention-to-treat population, comprising all participants receiving allocated test drug. In addition, we will conduct a pre-planned sub-analysis including all participants receiving allocated test drug but not requiring rescue drugs. We have added the sub-analysis to the fourth paragraph of the ‘Analysis plan’ as this was previously omitted by mistake.**

****

***Comment***

***“Laboratory tests.*** *…Normal values of troponin I in a female population (age 18 to 50 years) should be <15 ng/L, with the cut-off corresponding to the 99 percentile of a healthy reference population.* *17” *

- *In ref. 17 (Bahadur et al)* *^2^* *, the Pakistan population had a median age of the included female on 56 years old with the oldest being 86 and youngest being 21 years old. Most of the people 246 (82.3%) were above 40 years old. This means that they were older than the Norwegian CS population, and Troponin I increase with age, according to ref. 15, Venge et al.* *^3^* *Do the authors have any comments on this?*

- *Do we know if the Pakistan women have a similar Troponin level as Norwegian?*
*  *
- *What was the baseline level in the pilot study?*
*  *
- *Is Troponin I the best measurement for myocardial injury?*

**Author response**

**It is well known that level of high-sensitive troponins in healthy patients vary with age and sex. During recent years, the 99 ^th^ percentile cut-off for normal circulating concentration of high-sensitive troponin I (cTnI) has been lowered to <15 ng/L. This corresponds well to the findings of Kimenai et al. investigating 1540 individuals in a healthy European reference population (age 57±8 years, 52.4% women). Overall 99th percentile upper reference limits of cTnT and cTnI were 15 and 13 ng/L, respectively** **(Kimenai et al. 2016)** **. Bossard et al published 99 ^th^ percentile cut off for normal high-sensitive troponin I in a young healthy European population of 2077 adults from the general population aged 25–41 years without cardiovascular disease. The 99th percentile of cTnI was 15.79 ng/L in men and 5.11 ng/L in women** **(Bossard et al. 2017)** **. Kimenai et al. also found that cardiac troponin concentrations differ in women and men and are stronger predictors of cardiovascular events in women** **(Kimenai et al. 2021)** **.**

**The baseline level of cTnI in the pilot study was detected within the normal limits in all but one patient.**

**Recently Furenäs et al. published a study investigating dynamics of NTproBNP and cTnT in 196 healthy pregnant women. They found that existing cut-off values for ruling out heart failure (NTproBNP <300 ng/L) and myocardial ischaemia (hs-cTnT <14 ng/L) are applicable during pregnancy and after delivery, and that elevated levels mandate further attention on cardiac symptoms and renal function** **(Furenäs et al. 2020)** **. We have amended the text in section ‘Laboratory tests’ to explain these points and added additional supporting references.**

**High-sensitive troponin is the preferred tool for detecting myocardial stress and injury. In our clinic the lower detection limit of cTnT is 5 ng/L whereas levels of cTnI down to 0.1 ng/L were detected in our material. By choosing cTnI we are able to describe even small changes in high-sensitive troponin more accurately in a healthy population of young women.**

***Comment***

- *What about proBNP?*

**Author response**

**Dynamics of NTproBNP was investigated in the pilot trial. We found no group differences in the development of NTproBNP and for this reason we decided not to proceed with measurements of NTproBNP in the CMT2 trial.**

***Comment***

- *If all the levels are < the 99 ^th^percentile i.e. normal, can we then state that Carbetocin and Oxytocin are safe (regarding cardiac side-effects)?*
*  *
- *If there are minor differences but still within normal ranges, then this might not be clinically relevant? Like studies showing significant difference in blood loss on i.e., 178mL between treatment A and B...* *Significant - yes. Relevant - no!  Any comments?*

**Author response**

**We have reason to believe that even small elevations of high-sensitive troponins in relation to non-cardiac surgery are of clinical interest. Devereaux et al. investigated 21,842 adult patients undergoing non-cardiac surgery, mean age 63.1±10.7 years and 49.1% were female. They found that an absolute hsTnT change of 5 ng/L or higher was associated with an increased risk of 30-day mortality** **(Devereaux et al. 2017)** **. Although in this study participants were much older than in our study, we believe that transient elevations may indicate subclinical myocardial injury that may result in a higher risk of future cardiovascular event that may be treatable or preventable. We have not made any specific amendments to this comment, but refer to our earlier response that transient elevations in troponins after physical activity may be an early marker for cardiovascular events.**

***Comment***

***“Power and sample size considerations.*** *The sample size required for our current study was calculated using data from a pilot study of 40 patients, in which the ****largest difference in plasma troponin I concentration was found at 10 hours, with a mean +/- standard deviation change ****from baseline of 0.41+/-0.79 ng/L in the Carbetocin group versus 1.78+/-4.48 in the Oxytocin group”. *

- *I admit I am not used to doing statistical analysis, but does this mean that the change from the baseline can be 0.4 ng/L – 0.8ng/L = -0.4 ng/L? Because then Carbetocin improves the cardiac effect, reducing the Troponin I level?* *And similar for Oxytocin?*
*  *
- *Or does this mean that the data is skewed? And is “Median” not preferred instead of “Mean”?*

**Author response**

**The measurements of cTnI in our pilot study were skewed, and therefore best described by median and range, however for sample size calculations mean values and standard deviations are used.**

***Comment***

- *What is the baseline for Oxytocin and Carbetocin, respectively?*

**Author response**

**The baseline cTnI values for both the oxytocin and carbetocin groups in the Pilot study were similar.**

***Comment***

- *Is this because the authors suspect the results to be clinically not relevant, although detecting significant differences?*

**Author response**

**We started the pilot study not knowing whether there would be any difference between oxytocin and carbetocin in their cardiac effects but as oxytocin is known to affect the myocardium, we thought it was worth investigating given that carbetocin has a longer duration of action than oxytocin. As both oxytocin and carbetocin are used for PPH prophylaxis treatment, if it turns out that one has a less pronounced effect on the myocardium, this will help make clinical decisions for pregnant women with heart disease.**

*Comment*

- *And is this because Ferring is funding the project? (As I mentioned in the beginning, the reader should be more suspicious).*

**Author response**

**We have been transparent in the study’s funding and Ferring’s role in the study.**

***Comment***

***Analysis plan***

- *I believe I do not have enough knowledge to comment relevantly on the analysis plan.  *

***Strength and Limitations. **In 2019, a core outcome set was published for the prevention of postpartum hemorrhage to aid better comparison of clinical trials ensuring a minimum of outcomes to be reported…We will not assess breastfeeding or patient satisfaction with treatment, but will report pain and pain relief medication.” *

- *Why is this relevant to the study?*

**Author response**

**The core outcome set for the prevention of PPH has been established to allow easier comparison of clinical trials studying prevention of PPH. The core outcome set was published after our protocol was approved. The relevance to our study is that we are assessing seven out of nine recommended core outcomes, except for breastfeeding and patient satisfaction. The core outcome set includes intention and maintenance of breastfeeding as important patient-centred outcome as PPH may trigger a series of events that prevent or reduce a mother’s ability to fully breastfeed, particularly if the mother and baby are separated. It is also worth noting that breastfeeding releases oxytocin and therefore affects uterus tone but there is insufficient evidence for whether breastfeeding reduces PPH risk.**

****

***Comment***

- *Is the dose of Oxytocin 2.5 IU used elsewhere?*
*  *
- *The cardiovascular events that are described after oxytocin iv dose (ST-changes, hypotension, tachycardia), have they been found after 2.5 IU?*

**Author response**

**No one has yet published the cardiovascular effects of oxytocin 2.5 IU IV. We have investigated this dose in our pilot study and will add the citation once it has been published.**

*Comment*

- *Will data be released at the person level (for i.e. reviewer) in case of potential cardiac damage, and high Troponin I level during the CS?*

**Author response**

**We are batch assessing high-sensitivity troponin I levels after the last patient has completed the trial, which means that all patients will received standard of care. Follow-up is for 48 hours after their caesarean delivery. We will not know whether individual patients have high Troponin I levels during their hospital stay. Further studies will be required to determine risk of cardiovascular events due to PPH prophylaxis treatment, if there is indeed any future risk.**

***Comment***

- ***Is the rationale for, and objectives of, the study clearly described?***

*Partly*

- ***Is the study design appropriate for the research question?***

*Yes*

- ***Are sufficient details of the methods provided to allow replication by others?***

*Partly*

- ***Are the datasets clearly presented in a useable and accessible format?***

*Not applicable*

**Author response**

**No response necessary.**

**References**

 Aengevaeren, V. L., M. T. E. Hopman, P. D. Thompson, E. A. Bakker, K. P. George, D. H. J. Thijssen and T. M. H. Eijsvogels (2019). "Exercise-Induced Cardiac Troponin I Increase and Incident Mortality and Cardiovascular Events." Circulation **140**(10): 804-814.

 Bossard, M., S. Thériault, S. Aeschbacher, T. Schoen, S. Kunz, M. von Rotz, J. Estis, J. Todd, M. Risch, C. Mueller, L. Risch, G. Paré and D. Conen (2017). "Factors independently associated with cardiac troponin I levels in young and healthy adults from the general population." Clin Res Cardiol **106**(2): 96-104.

 Cole, N. M., I. Abushoshah, K. G. Fields, D. A. Carusi, J. N. Robinson, B. T. Bateman and M. K. Farber (2021). "The interrater reliability and agreement of a 0 to 10 uterine tone score in cesarean delivery." Am J Obstet Gynecol MFM **3**(3): 100342.

 Dell-Kuster, S., I. Hoesli, O. Lapaire, E. Seeberger, L. A. Steiner, H. C. Bucher and T. Girard (2017). "Efficacy and safety of carbetocin given as an intravenous bolus compared with short infusion for Caesarean section - double-blind, double-dummy, randomized controlled non-inferiority trial." Br J Anaesth **118**(5): 772-780.

 Devereaux, P. J., B. M. Biccard, A. Sigamani, D. Xavier, M. T. V. Chan, S. K. Srinathan, M. Walsh, V. Abraham, R. Pearse, C. Y. Wang, D. I. Sessler, A. Kurz, W. Szczeklik, O. Berwanger, J. C. Villar, G. Malaga, A. X. Garg, C. K. Chow, G. Ackland, A. Patel, F. K. Borges, E. P. Belley-Cote, E. Duceppe, J. Spence, V. Tandon, C. Williams, R. J. Sapsford, C. A. Polanczyk, M. Tiboni, P. Alonso-Coello, A. Faruqui, D. Heels-Ansdell, A. Lamy, R. Whitlock, Y. LeManach, P. S. Roshanov, M. McGillion, P. Kavsak, M. J. McQueen, L. Thabane, R. N. Rodseth, G. A. L. Buse, M. Bhandari, I. Garutti, M. J. Jacka, H. J. Schünemann, O. L. Cortes, P. Coriat, N. Dvirnik, F. Botto, S. Pettit, A. S. Jaffe and G. H. Guyatt (2017). "Association of Postoperative High-Sensitivity Troponin Levels With Myocardial Injury and 30-Day Mortality Among Patients Undergoing Noncardiac Surgery." Jama **317**(16): 1642-1651.

 Furenäs, E., P. Eriksson, U. B. Wennerholm and M. Dellborg (2020). "Pregnancy in a healthy population: dynamics of NTproBNP and hs-cTroponin T." Open Heart **7**(2).

 Gresslien, T. and S. Agewall (2016). "Troponin and exercise." Int J Cardiol **221**: 609-621.

 Heesen, M., B. Carvalho, J. C. A. Carvalho, J. J. Duvekot, R. A. Dyer, D. N. Lucas, N. McDonnell, S. Orbach-Zinger and S. M. Kinsella (2019). "International consensus statement on the use of uterotonic agents during caesarean section." Anaesthesia **74**(10): 1305-1319.

 Kimenai, D. M., R. M. Henry, C. J. van der Kallen, P. C. Dagnelie, M. T. Schram, C. D. Stehouwer, J. D. van Suijlen, M. Niens, O. Bekers, S. J. Sep, N. C. Schaper, M. P. van Dieijen-Visser and S. J. Meex (2016). "Direct comparison of clinical decision limits for cardiac troponin T and I." Heart **102**(8): 610-616.

 Kimenai, D. M., A. S. V. Shah, D. A. McAllister, K. K. Lee, A. Tsanas, S. J. R. Meex, D. J. Porteous, C. Hayward, A. Campbell, N. Sattar, N. L. Mills and P. Welsh (2021). "Sex Differences in Cardiac Troponin I and T and the Prediction of Cardiovascular Events in the General Population." Clin Chem **67**(10): 1351-1360.

 Rosseland, L. A., T. H. Hauge, G. Grindheim, A. Stubhaug and E. Langesaeter (2013). "Changes in blood pressure and cardiac output during cesarean delivery: the effects of oxytocin and carbetocin compared with placebo." Anesthesiology **119**(3): 541-551.

 Sarna, M. C., A. K. Soni, M. Gomez and N. E. Oriol (1997). "Intravenous oxytocin in patients undergoing elective cesarean section." Anesth Analg **84**(4): 753-756.

 Svanström, M. C., B. Biber, M. Hanes, G. Johansson, U. Näslund and E. M. Bålfors (2008). "Signs of myocardial ischaemia after injection of oxytocin: a randomized double-blind comparison of oxytocin and methylergometrine during Caesarean section." Br J Anaesth **100**(5): 683-689.

 Tabl, S., M. Balki, K. Downey, G. Tomlinson, D. Farine, G. Seaward and J. C. A. Carvalho (2019). "Uterotonics in elective caesarean delivery: a randomised non-inferiority study comparing carbetocin 20 μg and 100 μg." Anaesthesia **74**(2): 190-196.

 Thomas, J. S., S. H. Koh and G. M. Cooper (2007). "Haemodynamic effects of oxytocin given as i.v. bolus or infusion on women undergoing Caesarean section." Br J Anaesth **98**(1): 116-119.

# Peer review: Reviewer response for version 1

**DOI:** 10.5256/f1000research.76738.r95511

**Published:** 2021-10-26

**Authors:** Pamela J. Angle

**Affiliations:** 1 Sunnybrook Research Institute, Toronto, Canada

The protocol describes a parallel, blinded, two-arm randomized controlled safety trial which aims to examine the myocardial effects of intravenous carbetocin (100mcg ) versus oxytocin (2.5units), given over 1 minute immediately after elective cesarean delivery.  Rescue therapy will include the use of oxytocin as necessary in both groups (up to 5 units). The study will recruit 240 women, with single gestation pregnancies greater than 36 weeks, and without medical comorbidities  (e.g. hypertension, diabetes),  from three university-affiliated clinics in Norway. Women will receive standardized management of spinal anesthesia, the fluid co-load, and vasopressors. The primary endpoint will be changes in high sensitivity Troponin I levels, measured at baseline preoperatively and 6-10 hours after drug administration between groups as well as other important secondary outcomes, including severe adverse events, measured up to 48 hours after delivery. Differences in pain between groups will be assessed at one study site. Women will receive multimodal analgesia and intravenous patient-controlled opioid analgesia.  Given that oxytocin is a first line drug for the prevention of uterine atony and postpartum hemorrhage, the variability with which it is currently dosed, and the rising age and co-morbidities found in childbearing women in developed countries, study findings may have important implications for the management of patients at higher cardiac risk during cesarean section.  

 The Spirit checklist was previously (2018) completed by the authors during protocol development but some elements of the trial design and conduct remain unclear. Protocols available on other trial registry websites provide much of the additional information required. The study is funded, in part, by an unrestricted grant from the makers of Carbetocin. The authors note that funders had no role in study design, data collection, and analysis, decision to publish, or preparation of the manuscript.

 It should be made clear early in the protocol that the current safety Trial builds on the authors’ previous pilot work (randomized controlled trial, n=40) to provide the reader with more context. The pilot study demonstrated a reduction in changes in high sensitivity Troponin I levels from baseline with Carbetocin( 0.41+/-0.79ng/L ) compared with oxytocin (1.78 +/-4.48 ng/L) after elective cesarean delivery.  It also showed that changes in high sensitivity Troponin I peaked 10 hours after drug administration when the primary outcome of the current trial is to be measured.  [Protocol Synopsis, The Clinical Carbetocin Myocardium Trial- Part II ^1^]. Information provided in the sample size estimation section suggests that the study is powered as a superiority trial aimed at confirming findings of the previous pilot work.

 Additional details and clarification related to trial organization and how the trial is run from day to day at the three sites are required. The role of the clinical trials unit mentioned seems to be limited to the generation of the randomization schedule and oversight/monitoring of the trial itself.     

 Please provide additional information related to randomization (e.g. were random permutated blocks of different sizes used?  As reported, the person making up study drugs was the only person who knew treatment assignments. As reported, the PI screened potential candidates across all three sites for eligibility.  Who consented patients? How exactly were patients allocated to treatment arms?  Was this internet-based using Viedoc also?  Were baseline Troponin I levels available for any of the patients prior to treatment allocation or were these also batched with the 6-10 hour samples for testing?  Is there any plan to assess the blinding of the anesthetist involved in patient care during the c-section?

 The primary analysis will be by modified intention to treat. The primary outcome will be assessed using an independent student t-test followed by regression modeling. It appears the authors assume that randomization will balance any known confounders (e.g. women with hypertension, diabetes) between treatment arms. It will be important to assess whether any significant difference found in the primary outcome holds up after adjusting for imbalances.

Is the study design appropriate for the research question?

Yes

Is the rationale for, and objectives of, the study clearly described?

Partly

Are sufficient details of the methods provided to allow replication by others?

Yes

Are the datasets clearly presented in a useable and accessible format?

Not applicable

Reviewer Expertise:

Anesthesiology, Health Research Methodology, Obstetrical anesthesia

I confirm that I have read this submission and believe that I have an appropriate level of expertise to confirm that it is of an acceptable scientific standard.

## References

[1] : The Clinical Carbetocin Myocardium Trial – Part II. Reference source

# Response

**Published:** 2022-03-31

**Authors:** Leiv Arne Rosseland

**Affiliations:** Oslo University Hospital, Norway

PROTOCOL MANUSCRIPT - Review

*26 Oct 2021 | for Version 1*

**Pamela J. Angle**, Sunnybrook Research Institute, Toronto, Canada 

 Reviewer comments are in *italicized *text. Author responses are in **bold** text.

**Comment 1**

*The protocol describes a parallel, blinded, two-arm randomized controlled safety trial which aims to examine the myocardial effects of intravenous carbetocin (100mcg ) versus oxytocin (2.5units), given over 1 minute immediately after elective cesarean delivery.  Rescue therapy will include the use of oxytocin as necessary in both groups (up to 5 units). The study will recruit 240 women, with single gestation pregnancies greater than 36 weeks, and without medical comorbidities  (e.g. hypertension, diabetes),  from three university-affiliated clinics in Norway. Women will receive standardized management of spinal anesthesia, the fluid co-load, and vasopressors. The primary endpoint will be changes in high sensitivity Troponin I levels, measured at baseline preoperatively and 6-10 hours after drug administration between groups as well as other important secondary outcomes, including severe adverse events, measured up to 48 hours after delivery. Differences in pain between groups will be assessed at one study site. Women will receive multimodal analgesia and intravenous patient-controlled opioid analgesia.  Given that oxytocin is a first line drug for the prevention of uterine atony and postpartum hemorrhage, the variability with which it is currently dosed, and the rising age and co-morbidities found in childbearing women in developed countries, study findings may have important implications for the management of patients at higher cardiac risk during cesarean section.*

*The Spirit checklist was previously (2018) completed by the authors during protocol development, but some elements of the trial design and conduct remain unclear. Protocols available on other trial registry websites provide much of the additional information required. The study is funded, in part, by an unrestricted grant from the makers of Carbetocin. The authors note that funders had no role in study design, data collection, and analysis, decision to publish, or preparation of the manuscript.*

*It should be made clear early in the protocol that the current safety Trial builds on the authors’ previous pilot work (randomized controlled trial, n=40) to provide the reader with more context. The pilot study demonstrated a reduction in changes in high sensitivity Troponin I levels from baseline with Carbetocin( 0.41+/-0.79ng/L ) compared with oxytocin (1.78 +/-4.48 ng/L) after elective cesarean delivery.  It also showed that changes in high sensitivity Troponin I peaked 10 hours after drug administration when the primary outcome of the current trial is to be measured.  [Protocol Synopsis, The Clinical Carbetocin Myocardium Trial- Part II* *^1^* *]. Information provided in the sample size estimation section suggests that the study is powered as a superiority trial aimed at confirming findings of the previous pilot work.*

**Author response**

**We have added text to the ‘Aims and objectives’ to state that the protocol of this current study has followed a pilot study. The pilot study is not currently published as it is undergoing journal review.**

***Comment***

*Additional details and clarification related to trial organization and how the trial is run from day to day at the three sites are required. The role of the clinical trials unit mentioned seems to be limited to the generation of the randomization schedule and oversight/monitoring of the trial itself.*

**Author response**

**We have amended the text to state that the principal investigator from each study site screened women for inclusion in the study.**

***Comment***

*Please provide additional information related to randomization (e.g. were random permutated blocks of different sizes used?  As reported, the person making up study drugs was the only person who knew treatment assignments. As reported, the PI screened potential candidates across all three sites for eligibility.  Who consented patients? How exactly were patients allocated to treatment arms?  Was this internet-based using Viedoc also?  Were baseline Troponin I levels available for any of the patients prior to treatment allocation or were these also batched with the 6-10 hour samples for testing?  Is there any plan to assess the blinding of the anesthetist involved in patient care during the c-section?*

**Author response**

**We have amended the article to better describe the organization of randomization in Viedoc and assessment of blinding and allocation of patients. We have clarified that test assessing each responsible investigator will be performed in the ‘Randomization and blinding’ subsection.**

**Baseline troponin I levels were not assessed prior to randomization. All troponin I levels were batch assessed and none of the investigators will have access to these results until after the last patient has completed the study. No changes have been made with regard to assessment of troponin I levels as these are explained in the subsection ‘Laboratory tests’**

***Comment***

*The primary analysis will be by modified intention to treat. The primary outcome will be assessed using an independent student t-test followed by regression modeling. It appears the authors assume that randomization will balance any known confounders (e.g. women with hypertension, diabetes) between treatment arms. It will be important to assess whether any significant difference found in the primary outcome holds up after adjusting for imbalances.*

- ***Is the rationale for, and objectives of, the study clearly described?***

*Partly*

- ***Is the study design appropriate for the research question?***

*Yes*

- ***Are sufficient details of the methods provided to allow replication by others?***

*Yes*

- ***Are the datasets clearly presented in a useable and accessible format?***

*Not applicable*

**Author response**

**We have added a few sentences at the end of the ‘Introduction’ to provide more clearly the rationale for conducting the study. In addition, we have provided more information about the statistical analysis as we will conduct additional prospective sub-analysis of patients who did not receive rescue treatment in cases of uterine atony as this may change the results.**
